# Influencing medication taking behaviors using automated two‐way digital communication: A narrative synthesis systematic review informed by the Behavior Change Wheel

**DOI:** 10.1111/bjhp.12580

**Published:** 2022-01-26

**Authors:** Gemma Donovan, Nicola Hall, Jonathan Ling, Felicity Smith, Scott Wilkes

**Affiliations:** ^1^ Faculty of Health Sciences and Wellbeing School of Pharmacy and Pharmaceutical Sciences University of Sunderland UK; ^2^ Faculty of Medical Sciences Population Health Sciences Institute Newcastle University UK; ^3^ Faculty of Health Sciences and Wellbeing University of Sunderland UK; ^4^ UCL School of Pharmacy UK; ^5^ Faculty of Health Sciences and Wellbeing School of Medicine University of Sunderland UK

**Keywords:** treatment compliance, mobile health, behaviour change, chronic illness

## Abstract

**Purpose:**

Around half of prescribed medications for long‐term conditions are not taken as directed. Automated two‐way digital communication, such as text messaging and interactive voice response technology, could deliver interventions to improve medication adherence, and subsequently health. However, exploration of how such interventions may improve medication adherence is limited. This review aimed to explore how automated two‐way digital communication can improve medication taking with or without using non‐digital intervention components, such as phone calls with healthcare professionals.

**Methods:**

A theory‐informed narrative synthesis systematic review. Several databases were searched including CINAHL, Embase, Medline, and Web of Science using key words relating to ‘medication adherence’ and digital communication technologies. The Behavior Change Technique (BCT) coding using the BCT Taxonomy V1 and the Behavior Change Wheel were used to identify BCTs delivered within the included interventions.

**Results:**

A total of 3,018 records were screened with 43 study reports included in the review. Four medication‐taking behaviors: *taking medication*, *obtaining medication*, *self‐testing,* and *asking for support* were identified as targets for behavior change within the included interventions. Most BCTs within the digital communication component aimed to increase motivation for medication adherence, with non‐digital intervention components included to address other medication taking barriers, such as physical and psychological capability.

**Conclusion:**

Automated two‐way digital communication can detect barriers to medication adherence by monitoring performance of the *taking medication* behavior. Monitoring outcomes from *taking medication* may increase reflective motivation to take medicines. Addressing physical opportunity to *taking medication* by facilitating the behavior *obtaining medication* may also increase adherence.

**Statement of contribution**

***What is already known on this subject?***

- Around half of prescribed medications for long‐term conditions are not taken as directed.
- The Capability, Opportunity, and Motivation model of behavior has been used to explain medication‐taking.
- Automated two‐way digital communication has been shown to have positive effects on medication adherence.

***What does this study add?***

- Identification of four medication taking behaviors targeted using automated two‐way digital communication.
- Improved medication adherence was found in studies facilitating the behavior *obtaining medication*.
- Barriers to *taking medication* can be detected through behavioral monitoring using two‐way digital communication.

## Background

The increasing proportion of people living with long‐term conditions (LTCs) and multi‐morbidity is placing a growing burden on health and social care systems. Data from the UK Health Survey show that 45% of women and 41% of men have a longstanding illness (Fat, 2018). Medicines are the most common intervention to treat and manage LTCs; 48% of adults in the UK take at least one prescribed drug (Moody, Mindell, & Faulding, 2016). However, we know that 30–50% of patients with LTCs do not take their medication as directed by their prescriber (World Health Organization, 2003) and that evidence for interventions which tackle nonadherence effectively continues to be elusive. The impact of medication nonadherence is far reaching: a potential reduction in therapeutic effect can lead to patients requiring further intervention with subsequent economic implications (Cutler, Fernandez‐Llimos, Frommer, Benrimoj, & Garcia‐Cardenas, 2018). Improving medication adherence can decrease mortality rates in hypertensive patients (Morisky et al., 1983) and reduce hospitalisations in patients with asthma (Trautner, Richter, & Berger, 1993).

Reviews on the use of a wide range of digital communication technologies for supporting medication adherence have arrived at mixed conclusions. However, many reviews support their potential to enhance medication adherence (Fang, Maeder, & Bjering, 2016; Lee, Ralston, Beautrais, & Larkin, 2014; Sarabi, Sadoughi, Orak, & Bahaadinbeigy, 2016; Sarkar & Sivashankar, 2015; Vervloet et al., 2012) and that they are acceptable to patients (Anglada‐Martinez et al., 2015; Park, Howie‐Esquivel, & Dracup, 2014). A meta‐analysis has also found that text messages can improve adherence to medication (Thakkar, Kurup, & Laba, 2016). Some reviewers have also concluded that two‐way communication may be more effective than one‐way. Software can also be used to send messages automatically at appropriate times and respond to patients using pre‐set algorithms, providing the opportunity for cheap and low‐burden interventions (Iribarren, Brown, & Giguere, 2017). This review was intended for use as a basis to design a new intervention using such a system, Simple Telehealth (Simple Shared Healthcare Ltd, 2020). This uses Short Message Service (SMS) technolgy and was already available for use in the National Health Service (NHS). As such technology is relatively new, we expanded our review to examine evidence that could be adapted from older technologies, such as Interactive Voice Response (IVR) systems and pagers. However, we currently lack explanations as to *how* automated two‐way digital communication interventions might work to improve medication adherence.

Reasons for medication non‐adherence are also complex. Literature in this area describes a range of theories and models to explain and predict medicating taking behaviors (Easthall & Barnett, 2017). Behavioral frameworks, such as the Capability, Opportunity, and Motivation for Behavior (COM‐B) model (Michie, van Stralen, & West, 2011), can provide a comprehensive lens through which to examine the problem of medication nonadherence (Easthall & Barnett, 2017; Jackson, Eliasson, Barber, & Weinman, 2014). The COM‐B model describes behaviors as being influenced by an individual’s physical and psychological capability to perform a behavior, their physical and social opportunity to engage in the behavior, and their reflective and automatic motivation for conducting the behavior (Michie et al., 2011). The Behaviour Change Wheel (BCW) uses the COM‐B model as a framework for characterizing and designing behavior change interventions, including a mapping process to ‘intervention functions’ that then guide the selection of Behaviour Change Techniques (BCTs) (Michie, van Stralen, & West, 2011). A previous review has mapped medication taking to the COM‐B model (Jackson et al., 2014), and a more recent review mapped BCTs used in automated two‐way interventions for cardio‐metabolic conditions using meta‐regression (Kassavou & Sutton, 2018). Currently, a review which considers a full range of LTCs is lacking.

Text messaging alone is also unable to address more practical barriers to medication adherence, such as difficulties with patients accessing medicines from packaging. The use of digital communication may be optimised when used in addition to other medication‐related support (Ciciriello, Johnston, & Osborne, 2013; Fenerty, West, Davis, Kaplan, & Feldman, 2012; Granger & Bosworth, 2011; Hamine, Gerth‐Guyette, Faulx, Green, & Ginsburg, 2015; Mistry, Keepanasseril, & Wilczynski, 2015; Park, Howie‐Esquivel, & Dracup, 2014), such as communication with healthcare professionals in follow‐up telephone calls or face‐to‐face consultations. There are no reviews which consider these non‐digital intervention components, so it is currently unknown as to how these two elements may interact.

### Objectives

The aim of this review was to explore how two‐way automated digital communication interventions, with or without non‐digital components, can improve medication adherence and clinical outcomes for patient participants with long‐term conditions. This was achieved by 1) coding the BCTs, their target behavior, and delivery mode relating to medication adherence using intervention descriptions, 2) mapping the BCTs and behaviors to the most likely COM‐B component influenced for medication taking, and 3) comparing studied outcomes with the behavioral mechanism and delivery mode to identify how interventions may work to increase medication adherence and/or clinical outcomes for patients.

## Methods

We used a narrative synthesis systematic review method to evaluate the potential mechanisms of effectiveness for automated two‐way digital communication interventions on medication adherence and clinical outcomes using BCT coding and the BCW. A narrative synthesis review method was chosen as we anticipated high heterogeneity based on previous reviews (Kassavou & Sutton, 2018; Thakkar et al., 2016) and our broad inclusion criteria. This article focuses on one research question from a larger systematic review protocol registered on the PROSPERO Database prior to completion (CRD42017069290) and relates specifically to the use of the COM‐B model to characterise and explain the effects of automated two‐way patient contact interventions on medication adherence. The PRISMA statement 2020 (Page, McKenzie, & Bossuyt, 2020) was used to prepare this report, and the full checklist is available in the Supplementary Materials.

### Eligibility criteria

Studies were eligible for inclusion if their participants were adults (over 18 years) who were self‐managing their medication in their own home with any LTC, and the aim of the intervention was to affect medication adherence. Only interventions delivered in high‐income countries as classified by the World Bank (The World Bank, 2020) were considered to maximise transferability of findings into a new intervention in the UK NHS. The main intervention needed to include automated two‐way digital communication in its delivery. The digital communication technologies of interest were IVR or text messaging using SMS or pagers. Interventions could also have non‐digital intervention components in addition to the use of the automated two‐way digital communication. Any study comparator was considered. Studies were included if they reported outcomes relating to either medication adherence and/or clinical outcomes relevant to the LTC under study. Examples included clinical measurements such as blood pressure or symptom assessment tools. Only pilot or feasibility studies were excluded as these would not be powered to provide a robust evaluation of the outcome measures. Only studies published in English were examined. A full list of eligibility criteria can be found in Appendix S1.

### Information sources

Databases searched included Medline, CINAHL, PsycARTICLES, Psychology and Behavioral Sciences collection and International Pharmaceutical Abstracts using EBSCO Host, and Embase using Ovid. Web of Science, PubMed, and Cochrane Library were searched separately. Gray literature was also searched including the Simple Telehealth Network, British Library EthOS, and Trove and Opengrey.eu. The initial search was conducted in May–June 2017 with no lower date limit and was updated in September 2020. Reference lists from similar reviews were also evaluated for inclusion (Anglada‐Martinez et al., 2015; Ciciriello et al., 2013; Dekoekkoek, Given, & Given, 2015; Fang et al., 2016; Fenerty et al., 2012; Fjeldsoe, Marshall, & Miller, 2009; Granger & Bosworth, 2011; Hamine, Gerth‐Guyette, Faulx, Green, & Ginsburg, 2015; Lee et al., 2014; Linn, Vervloet, van Dijk, Smit, & Van Weert, 2011; Mistry et al., 2015; Nieuwlaat, Wilcyzynski, & Navarro, 2014; Park et al., 2014; Sarabi et al., 2016; Sarkar & Sivashankar, 2015; Tao, Xie, Wang, & Wang, 2015; Thakkar et al., 2016; Vervloet et al., 2012; Wald, Butt, Bestwick, & Bestwick, 2015). Supplementary documents to support BCT coding were obtained where possible, including published development studies and by contacting authors directly.

### Search

Thesaurus terms were used where available, supplemented by a range of key terms to search titles and abstracts. Search terms were developed to combine literature examining medication adherence and digital communication technologies. The selection and combination of terms was partially informed by similar reviews (Anglada‐Martinez et al., 2015; Ciciriello et al., 2013; Dekoekkoek et al., 2015; Fang et al., 2016; Fenerty et al., 2012; Fjeldsoe et al., 2009; Hamine et al., 2015; Lee et al., 2014; Linn et al., 2011; Mistry et al., 2015; Nieuwlaat et al., 2014; Park et al., 2014; Sarabi et al., 2016; Sarkar & Sivashankar, 2015; Tao et al., 2015; Thakkar et al., 2016; Vervloet et al., 2012; Wald et al., 2015). A full search strategy is available in Appendix S1.

### Study selection

Two reviewers (GD and NH) were involved in the initial study selection process. Titles and abstracts of the result list were screened independently by GD and NH to compile a list of articles for full text review. Discrepancies between the reviewers were discussed and agreed. Full text articles were then obtained where possible and again screened independently by GD and NH to create a list of studies for inclusion within the review. The updated search was conducted by GD based on discussions from the original study selection processes.

### Data collection processes

Data were extracted from each study on design characteristics, participant characteristics, intervention characteristics, intervention delivery details, and study outcomes. A data extraction tool was created using Google Forms (Google LLC, 2018) (see Appendix S2) and exported as an Excel spreadsheet to facilitate the comparison of inputs between both reviewers and the tabulation of results. The form was piloted by GD and then revised prior to extraction. Data entry was completed by GD and NH independently, with discrepancies resolved through discussion. Each study was assessed for quality using the Mixed‐Methods Appraisal Tool (MMAT) v1 (Pluye, Robert, Cargo, & Proposal, 2011). Data extraction for studies identified in the updated search was completed by GD, informed by the original discussions.

### Data analysis

Studies were first analyzed by describing study characteristics and outcomes. BCT coding was conducted for each individual study on multiple levels. Text describing the intervention was highlighted and coded for the inclusion of BCTs using the BCT Taxonomy v1 (Michie, Richardson, & Johnston, 2013), the behavior which the BCT targeted and the BCT mode of delivery (automated two‐way digital communication component or non‐digital component) in NVivo 12 (QSR International Pty Ltd. NVivo, 2018). This created ‘layers’ of coding which could be combined using the ‘overlapping’ search query function.

Coding was conducted independently by GD and NH, with differences resolved through discussion. A coding manual is available as Appendix S3. To support the coding of behaviors, potential medication self‐management behaviors were determined *a priori* by the study team based on experience as healthcare professionals and following guidance in the BCW (Michie, Atkins, & West, 2014). Using guidance in the BCW, the most likely intervention functions and COM‐B components that interventions seemed to use to influence medication taking were identified by the review authors, informed by study author explainations within journal articles where available. The identified BCW mechanisms were then charted against medication adherence and clinical outcomes to evaluate whether these mechanisms seemed to influence medication adherence and clinical improvements. This was done by considering the presence or absence of BCW mechanisms and whether outcomes from these interventions were improved, not improved, or had unclear results by vote counting the relevant included studies. Outcome categories were decided based on a combination of outcomes as reported in the original articles and consideration of the validity of outcomes in the context of study quality appraisal, in particular the method of medication adherence measurement. This is discussed as part of the results. The frequency with which BCTs were directed against the different behaviors was determined using searches of overlapping coding facilitated by NVivo 12 (QSR International Pty Ltd. NVivo, 2018). Analysis was completed separately for the automated digital communication and non‐digital intervention components to consider how these two delivery formats may be contributing to overall intervention outcomes.

## Results

### Study selection

**Figure 1:**
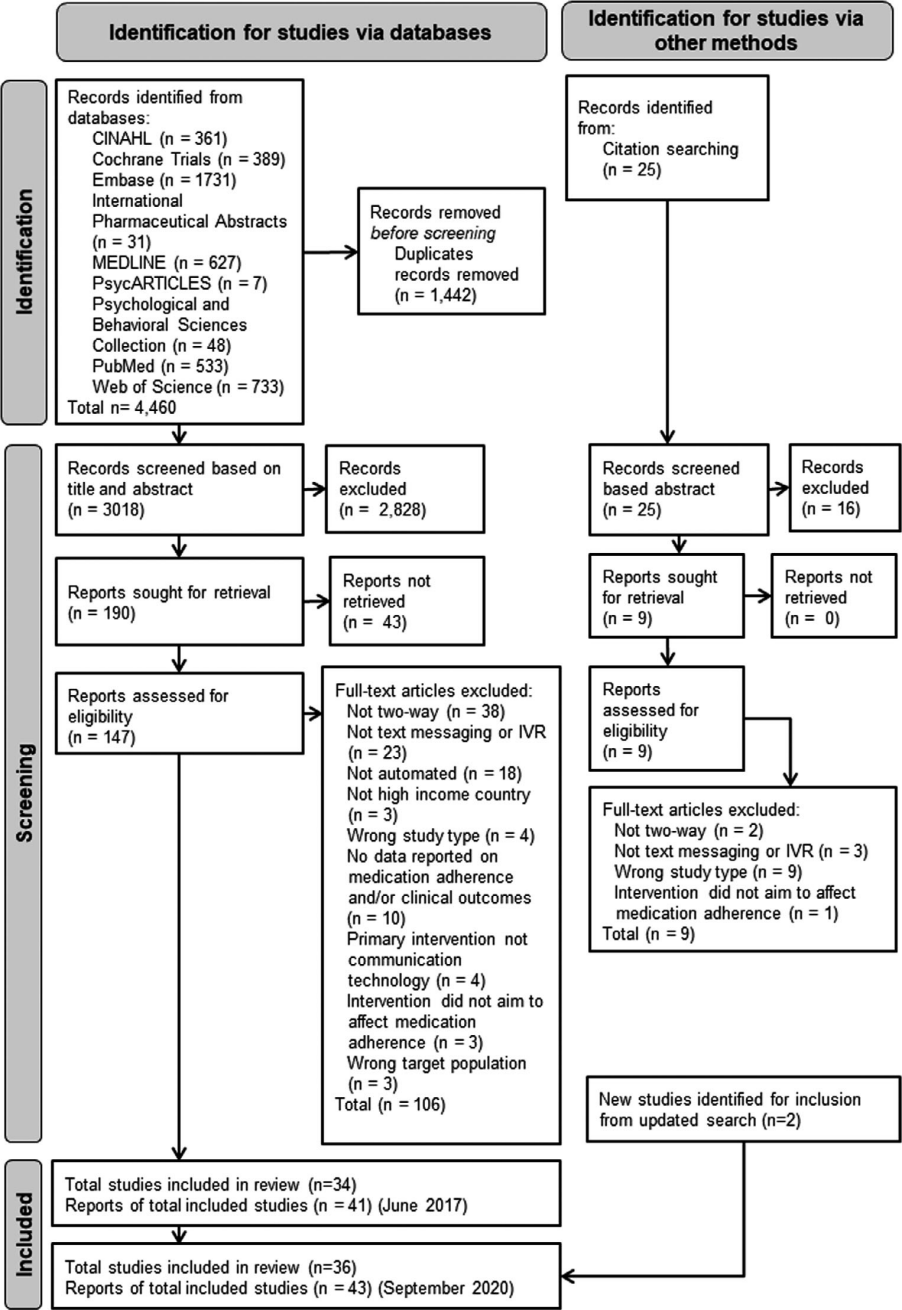
PRISMA Diagram representing selection of included studies.

A summary of the screening and filtering of search results for the original search is provided in the PRISMA diagram (Figure 1). No additional records were found using the gray literature, but a further 25 were found from the pearling of references from other published reviews. Forty‐one papers covering 34 studies were included from the original search. A further two studies and associated articles were identified from the updated search (Johnston, van der Kop, & Smillie, 2018; Kuusalo, Sokka‐Isler, & Kautiainen, 2020). Most of the studies examined the impact of interventions on medication adherence (*n* = 35) and 20 examined clinical outcomes.

### Study characteristics

A summary of the study characteristics can be found in Table 1. Most included studies were randomised controlled trials (n = 28). Studies were predominantly conducted in the United States (n = 30). The number of participants included within studies ranged from 40 to 21,752. A summary of the quality appraisal using the MMAT can be found in Appendix S4. Most of the RCTs were of good quality, usually only missing the ‘lack of allocation concealment’ criteria. Patient concealment is not possible with this type of intervention; however, some described concealment of investigators.

**Table 1:** Summary of included studies

Authors (Date)	Study design	Number of participants	Country	Comparator	Medication adherence outcome measure (where used)	Clinical outcome measure (where used)
Aikens, Trivedi, Heapy, et al. (2015)	Non‐randomised controlled trial	221	United States	IVR plus support person	MMAS	Depression remission (PHQ‐9 score of <5)
Aikens, Rosland, et al. (2015), Aikens, Trivedi, Aron, et al. (2015)	Non‐randomised controlled trial	303	United States	IVR with CarePartner (support person)	Self‐report *via* IVR, MMAS	Not studied
Bender et al. (2010)	Randomised controlled trial	50	United States	Usual care	Electronic tracking device on inhaler	Asthma Control Test
Boker et al., (2012)	Randomised controlled trial	40	United States	Usual care	MEMS cap on medication tube	Acne lesion counts, IGA Score
Boland et al. (2014)	Randomised controlled trial	70	United States	Usual care	Eye drops placed in a medicines bottle with MEMS cap	Not studied
Bove et al. (2013)	Randomised controlled trial	241	United States	Usual care	Medication self‐efficacy scale for hypertension in African Americans	BP, Cholesterol, BMI, Fasting BG, Triglycerides
Cizmic et al. (2015)	Randomised controlled trial	245	United States	Usual care	Medication Possession Ratio	Not studied
Friedman et al. (1996)	Randomised controlled trial	267	United States	Usual care	Pill count	BP
Garofalo et al. (2016)	Randomised controlled trial	109	United States	Baseline education about antiretroviral therapy without SMS reminders	Visual analog scale	Viral load
Glanz, Beck, & Bundy (2012)	Randomised controlled trial	312	United States	Usual care	Self‐report for medication taking and refills	Not studied
Harris et al. (2010), Simoni et al. (2009) and Yard et al. (2011)	Randomised controlled trial	224	United States	Usual care	MEMS and Simplified Medication Adherence Questionnaire	Viral load and CD4 count
Johnston et al. (2018)	Randomised controlled trial	358	Canada	Usual care	Self‐report or pill count (whichever was lowest)	Not studied
Katalenich et al. (2015)	Randomised controlled trial	98	United States	Usual care	MMAS	HbA1c
King et al. (2017)	Cohort study	85	Canada	Not applicable	Self‐report or prescription refill (whichever was lowest)	Viral load and CD4 Count
Kuusalo et al. (2020)	Randomised controlled trial	166	Finland	Usual care	Visual analog scale of treatment confidence	Rheumatoid arthritis remission
Leu et al. (2005)	Randomised controlled trial	50	United States	Usual care	Not studied	HbA1C and BP
Magid et al. (2011)	Randomised controlled trial	338	United States	Usual care	Medication Possession Ratio	Systolic BP, Diastolic BP
Mayberry et al. (2017)	Cohort study	80	United States	Not applicable	Summary of Diabetes self‐care activities medication subscale	HbA1C
Moore et al. (2015)	Randomised controlled trial	58	United States	Daily mood enquiries only.	MEMS	Not studied
Nelson, Mulvaney, Gebretsadik, Ho, et al., (2016), Nelson, Mulvaney, Gebretsadik, Johnson, et al. (2016)	Case‐control study	80	United States	Controls selected based on race, gender, and glycemic control and comparing HbA1c data at 3 months	Summary of Diabetes self‐care activities medication subscale	HbA1c
Nundy et al. (2013), (2014)	Cohort study	74	United States	Not applicable	MMAS	Not studied
Park, Howie‐Esquivel, Chung, et al. (2014), Park et al. (2015)	Randomised controlled trial	90	United States	TM with reminders + Education; Education TM only; No TM	MEMS	Not studied
Pfaeffli Dale et al. (2015)	Randomised controlled trial	123	New Zealand	Usual care	MMAS	BP, cholesterol, BMI, waist‐to‐hip ratio
Piette et al. (2000)	Randomised controlled trial	280	United States	Usual care	Medication 'problem' reporting via IVR assessment	HbA1c
Piette et al. (2015)	Randomised controlled trial	372	United States	Intervention plus Care Partner (support person)	Self‐report *via* IVR	Not studied
Shane‐McWhorter et al. (2014)	Cohort study	125	United States	Not applicable	MMAS	BP, HbA1c, Fasting lipids, BMI
Sherrard et al. (2009)	Randomised controlled trial	331	Canada	Usual care	Self‐report *via* IVR (question unclear)	Not studied
Sherrard et al. (2015)	Randomised controlled trial	1,608	Canada	Usual care	Self‐report *via* IVR (question unclear)	Not studied
Spoelstra et al. (2016)	Randomised controlled trial	75	United States	Usual care	Self‐report covering the last 7 days	Symptom Inventory
Stacy et al. (2009)	Randomised controlled trial	497	United States	Non tailored advice from 1 IVR call and print material	Possession of prescription	Not studied
Stuart et al. (2003)	Randomised controlled trial	647	United States	Group 1: Treatment team education and self‐care education; Group 2: education and call (1 office nurse call within 2 days of visit); Group 3: Education call and IVR (as group 2 plus IVR program lasting 3 months)	Self‐report via IVR (question unclear)	Not studied
Tucker, Simpson, Huang, Roth, & Stewart (2013)	Cohort study	44	United States	Not applicable	Self‐report in the previous 24 hours	Not studied
Vollmer, Feldstein, & Smith (2011)	Randomised controlled trial	8,517	United States	Usual care	Medication Possession Ratio	Asthma Therapy Assessment Questionnaire
Vollmer et al. (2014)	Randomised controlled trial	21,752	United States	Usual care and an arm with additional educational components including printed materials and a pill box	Modified PDC	LDL and Sysolic BP
Wald et al. (2014)	Randomised controlled trial	303	United Kingdom	Usual care	Self‐report covering the last 28 days	Not studied
Zabinski, Skinner, & Buysman (2012)	Cohort study	276 (IVR group)	United States	Medication adherence letter and non‐participants (usual care)	PDC	Not studied

Note

BG = Blood Glucose; BMI = Body Mass Index; BMQ = Beliefs about Medicines Questionnaire; BP = Blood Pressure; HbA1c = Glycosylated Haemoglobin; HDL = High‐Density Lipoprotein; IGA = Investigator Global Assessment; IVR = Interactive Voice Response; LDL = Low‐Density Lipoprotein; MEMS = Medication Event Monitoring System; MMAS = Morisky Medication Adherence Scale; mPDC = modified Proportion of Days Covered; PHQ = Patient Health Questionnaire; SMS = Short Message Service; TM = Text Messaging.

### Intervention characteristics

A summary of the intervention characteristics and detailed results can be found in the spreadsheet Appendix S5. The automated two‐way digital communication technologies examined included IVR (n = 19), SMS (n = 11), and pager devices (n = 2). Four studies used a combination of technologies, either to complement each other or to offer patient participants a choice on which mode of delivery they would prefer. Fifteen studies included at least one non‐digital intervention component alongside the use of automated two‐way digital communication.

Length of intervention ranged from a one‐off interactive call to 12 months of two‐way communication. The most common intervention aims were to improve adherence to an existing therapy (n = 23), followed by promoting adherence to a new therapy (n = 11). A small number of studies aimed to detect non‐adherence to medicines (n = 2) or maintain adherence to medication (n = 1). In one case, the aim of the intervention in relation to affecting medication adherence was unclear.

Most studies delivered interventions for a single LTC. The most common of these was cardiovascular disease (n = 13). Other LTCs included diabetes (n = 8), HIV/ AIDS (n = 6), depressive disorders (n = 4), osteoporosis (n = 1), cancer (n = 2), asthma (n = 2), chronic obstructive pulmonary disease (n = 1), tuberculosis (n = 1), glaucoma (n = 2), rheumatoid arthritis (n = 1), and acne (n = 1).

### Study results

Of the 36 studies included in the review, most (n = 34) provided outcomes for medication adherence and of these, 19 found improvement. Seven studies had unclear findings on medication adherence, and eight found no improvement. Two studies only assessed clinical outcomes, and an additional 18 provided clinical outcome data alongside medication adherence. Clinical outcomes were improved in seven studies but mostlu either did not find an improvement (n = 9) or had unclear outcomes (n = 4), even if there were improvements in medication adherence. Only four studies reported clearly positive outcomes in medication adherence and clinical outcomes.

### A medication behavioral map

**Figure 2:**
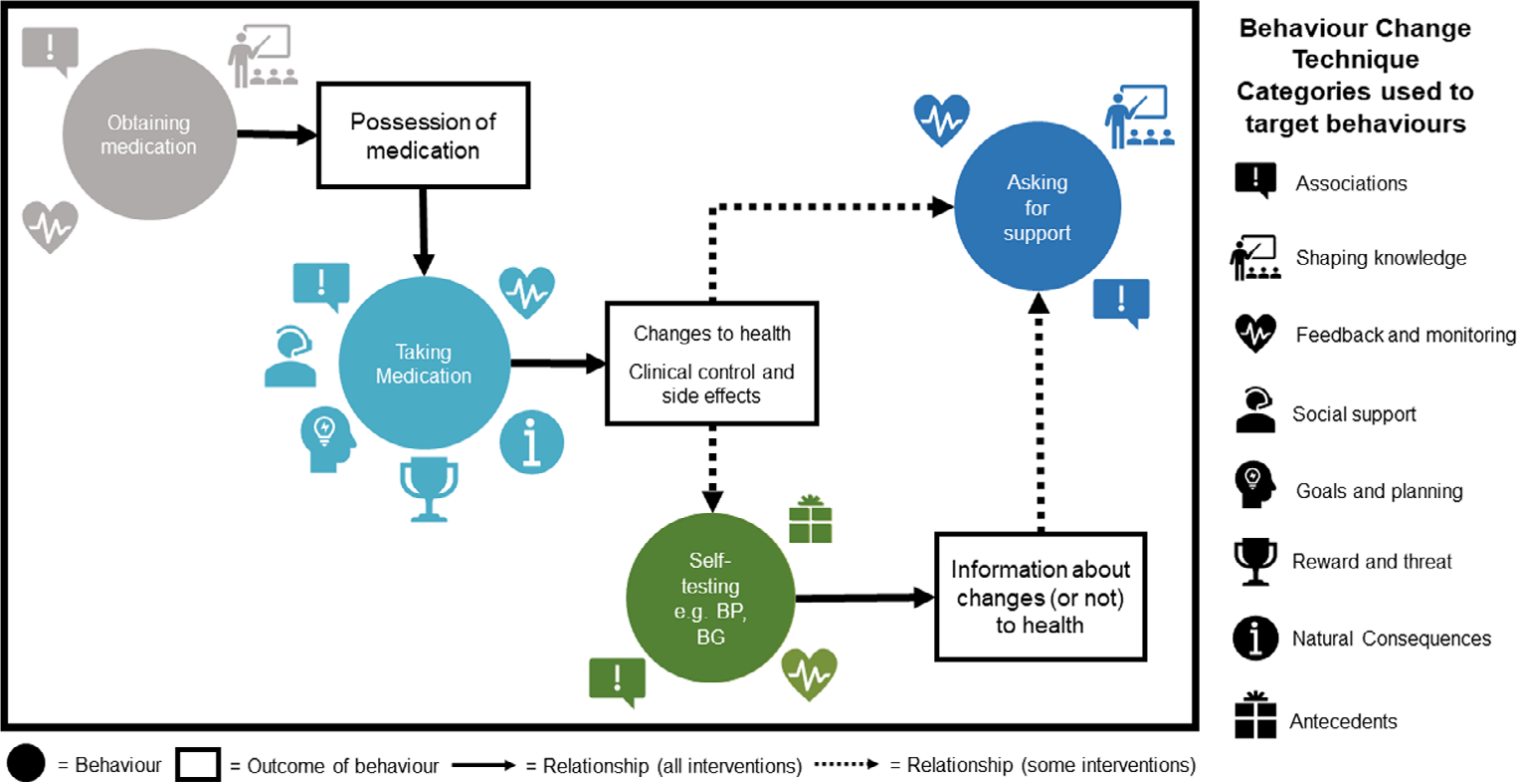
A map of the behaviors targeted within interventions and the Behavior Change Technique (BCT) categories used to target these within included interventions.

A summary of the behaviors targeted, the BCTs which were used, the delivery format of the BCTs, and outcomes for the included studies is available in the spreadsheet Appendix S5. Four medication‐related behaviors were targeted for change within interventions: *taking medication* (n = 35 studies); *obtaining medication* (n = 10); *self‐testing* (n = 9); and *asking for support* (n = 7). We describe the relationships between these behaviors in Figure 2 with a behavioral map. While there was often little detail about the support patients asked for, or what was delivered as the interventions included in the review all aimed to affect medication adherence, we have inferred that the behaviur *asking for support* would be linked to this outcome. Seventeen studies targeted just one behavior, 13 targeted two behaviors, and six targeted three behaviors.

**Figure 3:**
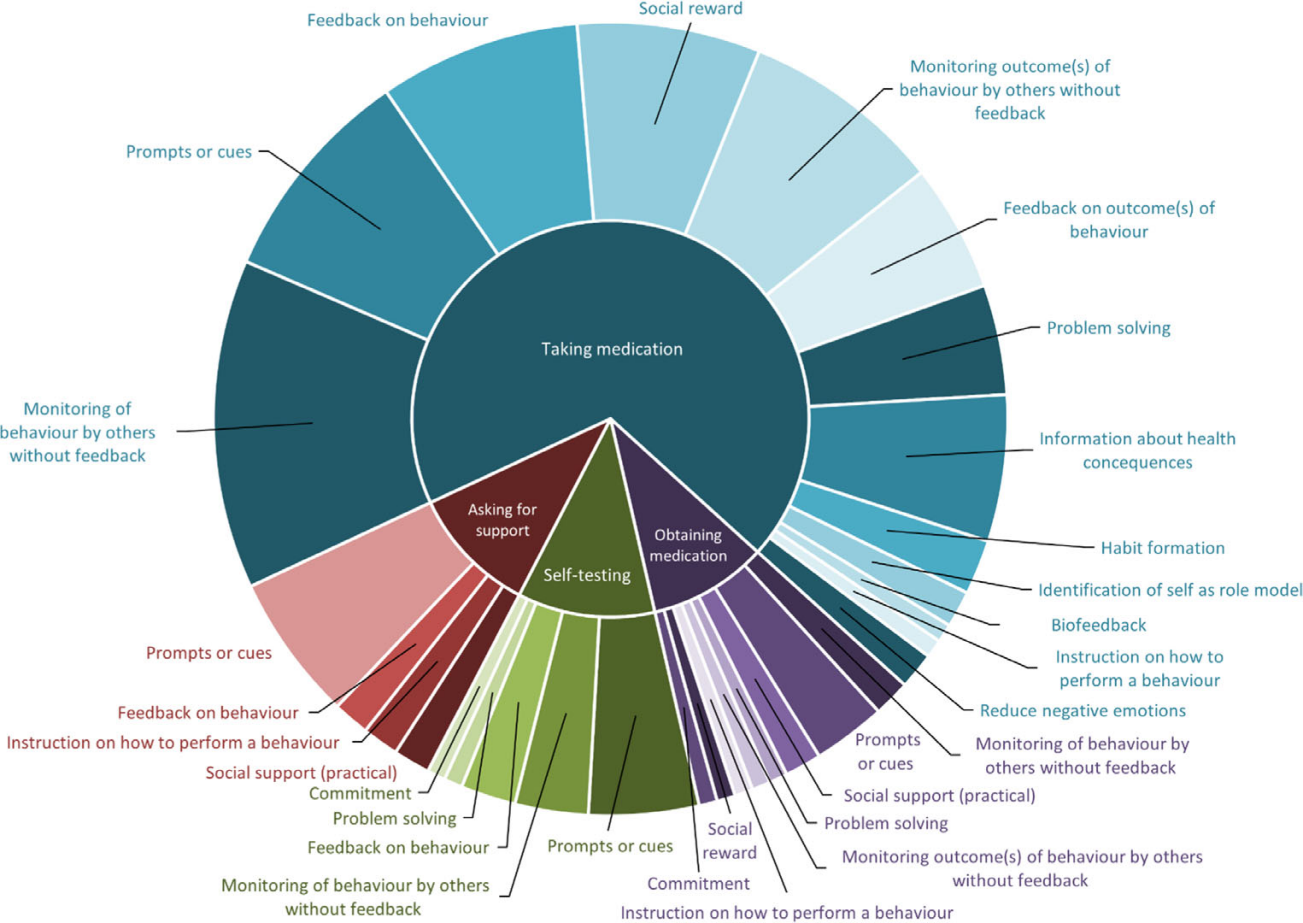
Sunburst diagram representing the frequency of Behavior Change Technique delivery against medication taking behaviors for automated two‐way digital communication intervention components.

**Figure 4:**
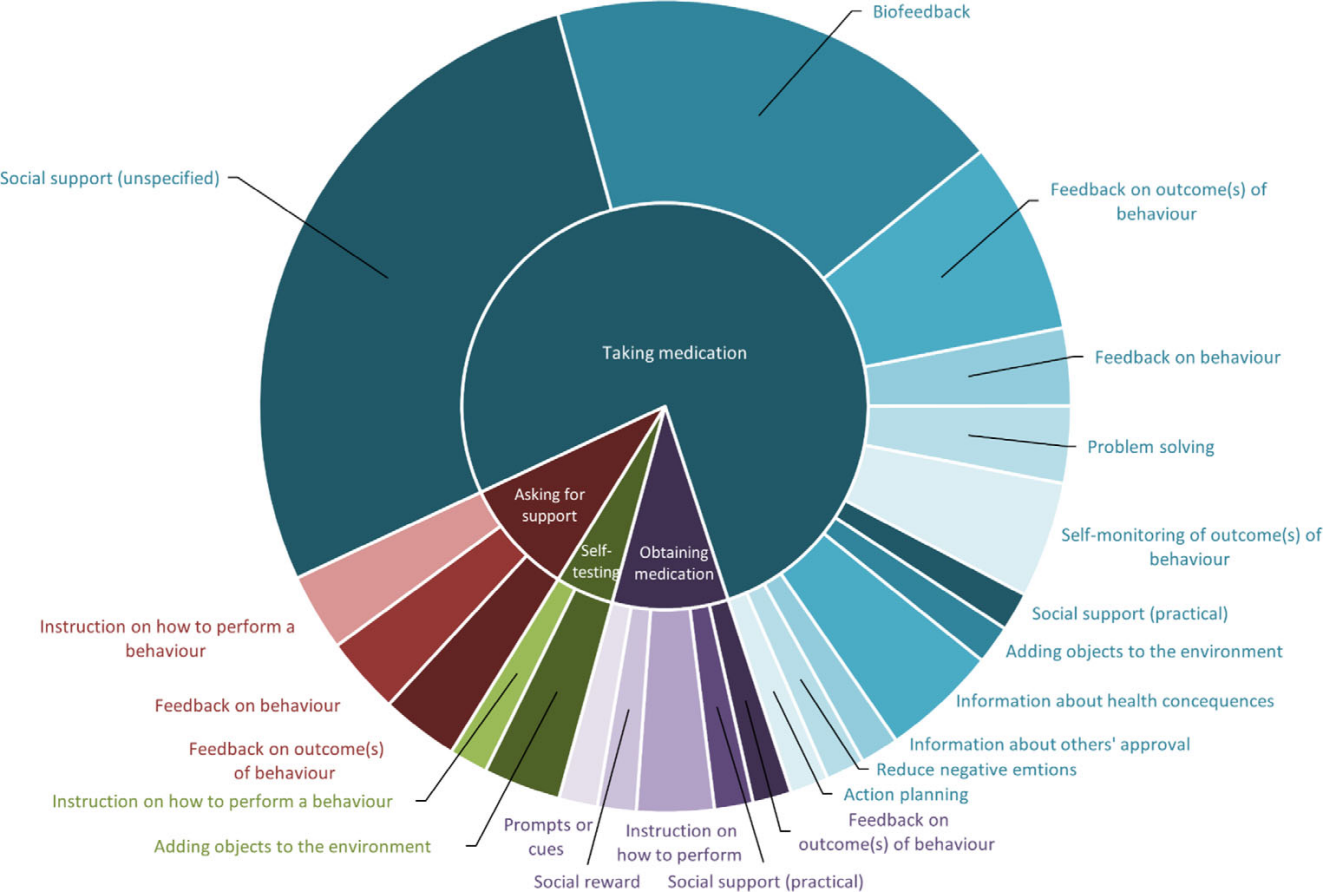
Sunburst diagram representing the frequency of Behavior Change Technique delivery against medication taking behaviors for non‐digital communication intervention components.

The number of BCTs included within interventions ranged from 1 to 10; however, there did not seem to be a relationship between the number of BCTs included and study outcomes. The average number of BCTs included in interventions with improvement in medication adherence was 4.8 versus 4.6 in those without improvement, and this was 5.6 BCTs in both groups for clinical outcomes. The most frequently included BCTs were *Prompts/Cues* (n = 19) and *Monitoring of behavior by others without feedback* (n = 19), which were mostly included within the automated digital communication component. Figure 3 summarises the number of study interventions targeting each medication‐taking behavior and the BCTs used for the automated digital communication component of interventions. *Social support (unspecified)* (n = 17) and *Biofeedback* (n = 12) were the most common BCTs included as part the non‐digital intervention components (see Figure 4).

There were also examples of integration between the automated two‐way digital communication and non‐digital components. This included interventions delivering different BCTs using different components or delivering the same BCT using both components. This has been highlighted in the color coding in Appendix S5. The following results are organised into how the behavioral components of interventions seemed to influence capability, opportunity, and motivation for the different medication taking behaviors identified.

#### Obtaining medication to remove the physical opportunity barrier to taking medication

Within our behavioral map (see Figure 2), performing the behavior *obtaining medication* is a pre‐requisite to the behavior *taking medication*. Without physical opportunity, *taking medication* is unlikely to occur. This behavior was targeted by both digital communication and non‐digital components (see Figure 3 and Figure 4). BCTs were identified as targeting *obtaining medication* where interventions influenced activities such as ordering medication from a prescriber and/or collecting it from a pharmacy. The most common BCT used to target this behavior was *Instruction on how to perform a behavior* using automated digital communication. Interventions that included BCTs to target *obtaining medication* had an almost universally positive impact on medication adherence with 90% of studies targeting this behavior finding improvements. This suggests that this behavior is a good target where physical opportunity is a barrier to taking medication.

#### Increasing automatic motivation to improve taking medication

Interventions which targeted *taking medication* in our behavioral map (see Figure 2) are those targeting self‐administering a medication. Interventions which targeted this behavior seemed to aim to improve medication adherence by supporting the formation of habit *via* automatic motivation. Although the *Habit formation* BCT has a narrow definition in the BCT Taxonomy v1, the repetition required to form a habit could be encouraged using a range of BCTs. For example, the BCT *Prompts/Cues* was frequently used to target *taking medication* with one‐way automated messages. An example message would be, “It’s time to take your medication”. Eleven studies include the use of one‐way *Prompts/Cues* (Boker, Feetham, Armstrong, Purcell, & Jacobe, 2012; Boland et al., 2014; Garofalo et al., 2016; Harris, Lehavot, & Huh, 2010; Katalenich, Shi, & Liu, 2015; Leu, Norris, Hummel, Isaac, & Brogan, 2005; Moore, Poquette, & Casaletto, 2015; Nundy, Dick, Solomon, & Peek, 2013; Nundy et al., 2014; Park, Howie‐Esquivel, Chung, & Dracup, 2014; Park, Howie‐Esquivel, Whooley, & Dracup, 2015; Simoni, Huh, & Frick, 2009; Spoelstra, Given, & Sikorskii, 2016; Wald, Bestwick, Raiman, Brendell, & Wald, 2014; Yard, Huh, King, & Simoni, 2011). However, the inclusion of this BCT did not have a consistent effect on medication adherence outcomes with only five of these reporting improvements in medication adherence.

Increasing awareness of the medication taking behavior through medication monitoring with or without feedback may have also been used to increase the medication taking habit. Monitoring behavior and providing feedback was automated in some studies (Aikens, Rosland, & Piette, 2015; Aikens, Trivedi, Aron, & Piette, 2015; Garofalo et al., 2016; Mayberry, Mulvaney, & Johnson, 2017; Moore et al., 2015; Piette, Weinberger, McPhee, Mah, & Kraemer, 2000; Sherrard, Duchesne, Wells, Kearns, & Struthers, 2015; Stuart, Laraia, Ornstein, & Nietert, 2003) while others used non‐digital components, such as follow‐up calls, to provide feedback (Leu et al., 2005; Sherrard, Struthers, Kearns, Wells, & Mesana, 2009), and two studies used a combination (Aikens, Trivedi, Heapy, Pfeiffer, & Piette, 2015; Nundy et al., 2013, 2014). There was evidence that providing feedback on behavior seemed to be slightly more linked to results for improved medication adherence, with 43% of studies providing feedback finding improvements in medication adherence compared to 38% of studies which monitored medication taking without feedback. Some studies also included the BCT *Social reward* with 60% of studies including this BCT (Aikens, Trivedi, Heapy, et al., 2015; Garofalo et al., 2016; Mayberry et al., 2017; Nundy et al., 2013, 2014; Piette et al., 2000; Stacy, Schwartz, Ershoff, & Shreve, 2009) reported an improvement in medication adherence.

Five studies used a combination of both the feedback and no feedback BCTs, usually with feedback only provided where medication nonadherence was identified. Four of these studies found improvements in medication adherence (Aikens, Rosland, et al., 2015; Aikens, Trivedi, Aron, et al., 2015; Nundy et al., 2013, 2014; Sherrard et al., 2009, 2015). This ability to detect nonadherence and follow‐up with patients may, therefore, be beneficial. A small number of studies (n = 8) also delivered the BCT *Problem Solving* (Aikens, Rosland, et al., 2015; Aikens, Trivedi, Aron, et al., 2015; Mayberry et al., 2017; Nelson, Mulvaney, Gebretsadik, Ho, et al., 2016; Nelson, Mulvaney, Gebretsadik, Johnson, & Osborn, 2016). All except the study by Aikens, Trivedi, Heapy, et al. (2015) reported improvements in medication adherence.

#### Increasing reflective motivation to improve medication taking

Interventions often seemed to aim to increase reflective motivation by attempting to persuade patient participants that medication taking is a ‘good’ thing to do. This was attempted in eight studies using one‐way communication delivering the BCT *Information about health consequences* directed at the behavior *taking medication* (Bender, Apter, & Bogen, 2010; Cizmic, Heilmann, Milchak, Riggs, & Billups, 2015; Friedman, Kazis, & Jette, 1996; Mayberry et al., 2017; Moore et al., 2015; Nelson, Mulvaney, Gebretsadik, Ho, et al., 2016; Nelson, Mulvaney, Gebretsadik, Johnson, et al., 2016; Pfaeffli Dale et al., 2015; Spoelstra et al., 2016). In all cases, this was delivered not only using the digital communication component but also supplemented *via* non‐digital intervention components, such as face‐to‐face consultations (Cizmic et al., 2015; Pfaeffli Dale et al., 2015; Spoelstra et al., 2016). As medicines can have both positive (health improvement) and negative (side effects) consequences, we have differentiated these in the online Supplementary Materials. Including this BCT did not, however, seem to have an effect on study outcomes with only 50% of studies reporting an improvement in medication adherence (Bender et al., 2010; Cizmic et al., 2015; Friedman et al., 1996; Mayberry et al., 2017).

Asking patients to actively monitor the health effects of taking medication could be used to influence their reflective motivation for medicine taking. For some LTCs, this monitoring requires the introduction of another behavior, which we have called *self‐testing* (see Figure 2). Examples included in this review were blood pressure (BP) or blood glucose (BG) home testing. Completing this *self‐testing* was coded as the *Biofeedback* BCT and was incorporated into 12 of our included studies (Aikens, Rosland, et al., 2015; Aikens, Trivedi, Aron, et al., 2015; Bove et al., 2013; Katalenich et al., 2015; Leu et al., 2005; Magid, Ho, & Olson, 2011; Nelson, Mulvaney, Gebretsadik, Ho, et al., 2016; Nelson, Mulvaney, Gebretsadik, Johnson, et al., 2016; Nundy et al., 2013, 2014; Piette, Striplin, & Marinec, 2015; Piette et al., 2000; Shane‐McWhorter, Lenert, & Petersen, 2014; Vollmer, Owen‐Smith, & Tom, 2014). Where *self‐testing* required the use of a device (e.g. blood glucose monitor), then this was classed as a non‐digital intervention component; however, three studies used automated two‐way digital communication to directly assess outcomes of *taking medication* using symptom assessment for depression (Aikens, Trivedi, Heapy, et al., 2015), asthma (Bender et al., 2010), and rheumatoid arthritis (Kuusalo et al., 2020).

Some interventions combined the use of *Biofeedback* with the BCT *Self‐monitoring of the outcomes of behavior (*Aikens, Rosland, et al., 2015; Aikens, Trivedi, Aron, et al., 2015; Nundy et al., 2013, 2014; Piette et al., 2000). This was delivered where patients were asked to keep records of home monitoring and only submit summaries of these *via* digital communication. As these self‐monitoring records were not submitted directly, we classified this BCT as delivered through a non‐digital component. However, digital communication was used to *Prompt/Cue* completion of the *self‐testing* behavior and monitor its completion in five studies (Katalenich et al., 2015; Leu et al., 2005; Magid et al., 2011; Nundy et al., 2013, 2014; Shane‐McWhorter et al., 2014), although examining the impact on the self‐testing behavior itself was not within the scope of this review.

The results from *self‐testing* were used in five studies to deliver the BCT *Monitoring outcome(s) of behavior by others without feedback*, (Friedman et al., 1996; Moore et al., 2015; Piette et al., 2000, 2015; Shane‐McWhorter et al., 2014) or *Feedback on the outcomes of behavior* in three studies (Bove et al., 2013; Magid et al., 2011; Vollmer et al., 2014). In the study by Katalenich et al. (2015) patient participants only received feedback if the results of the self‐testing required action, otherwise they were monitored without feedback. This was also the case for the assessment of depressive symptoms in the studies by Aikens, Trivedi, Heapy, et al. (2015) and Kuusalo et al. (2020). In these cases, *Monitoring of outcomes without feedback* seemed to be for the benefit of clinicians as part of remote monitoring rather than being used to support reflective motivation of patients for *taking medication*. Only the study by Bender et al. (2010) delivered feedback on outcomes *via* automated two‐way digital communication.

Of the five studies which only monitored outcomes without feedback, two reported improvements in medication adherence (Friedman et al., 1996; Piette et al., 2000), although an additional study reported improvements in clinical outcomes (Shane‐McWhorter et al., 2014). All of these studies were for patient participants with diabetes. Of the three studies which only used *Feedback on outcomes of behavior*, (Bove et al., 2013; Magid et al., 2011; Vollmer et al., 2014) one found an improvement in medication adherence (Vollmer et al., 2014) and one in clinical outcomes (Magid et al., 2011). All three studies were for patient participants with cardiovascular disease. Of the five studies using a combination of both BCTs, three found an improvement in medication adherence, (Aikens, Rosland, et al., 2015; Aikens, Trivedi, Aron, et al., 2015; Bender et al., 2010; Katalenich et al., 2015) with the study by Katalenich et al. (2015) also finding an improvement in diabetes control, but neither result was statistically significant. All three studies coded as including the *Self‐monitoring of outcomes of behavior* BCT found improvements in medication adherence. However, it should be noted that only the study by Bender et al. (2010) explicitly seemed to link these health outcomes with *taking medication*; most of the interventions studied only described linking these outcomes to lifestyle influences, such as diet or exercise. Where *Feedback on the outcomes of behavior* was delivered using a non‐digital component, insufficient detail was provided to know what exactly was discussed.

#### Removing psychological and physical capability barriers to taking medication

Providing an automated two‐way digital communication intervention has the potential to highlight psychological and physical capability barriers to *taking medication*. Including some form of ‘live’ interaction with a healthcare professional provides opportunities for patients to ask questions and healthcare professionals to assess the presence of these barriers. Where there was the opportunity for patient participants to have this assessment in a ‘live’ interaction, this was coded as the BCT *Social support (unspecified)* for the *taking medication* behavior. Seventeen studies included this BCT. However, details of what support was provided was often poorly described so coding further BCTs was not always possible. Five studies did describe providing some form of education to patient participants (Nundy et al., 2013, 2014; Piette et al., 2000; Shane‐McWhorter et al., 2014; Sherrard et al., 2015; Spoelstra et al., 2016), which could have addressed psychological capability barriers and two referenced improving motivation (Bove et al., 2013; Nundy et al., 2013, 2014). However, most referenced identifying ‘problems’ or ‘barriers’ to ‘self‐management’ which could potentially include a wide range of medication taking issues.

#### Asking for support to address psychological and physical capability gaps

Six studies used the BCT *Prompts/Cues* to trigger a new behavior from patients which we categorised as *asking for support* (Aikens, Trivedi, Heapy, et al., 2015; Bender et al., 2010; Johnston et al., 2018; King, Kinvig, & Steif, 2017; Magid et al., 2011; Nelson, Mulvaney, Gebretsadik, Ho, et al., 2016; Nelson, Mulvaney, Gebretsadik, Johnson, et al., 2016). Two studies also used digital communication to deliver the BCT *Social support (practical)* for *asking for support* by transferring the patient directly to a support line (Aikens, Trivedi, Heapy, et al., 2015) or linking this to a call‐back request, (Magid et al., 2011) both using IVR technology. However, targeting the *asking for support* behavior had no clear effect on medication adherence with only three out of the seven studies targeting this behavior reporting an improvement in medication adherence (Bender et al., 2010; Cizmic et al., 2015; King et al., 2017) and one in clinical outcomes (Magid et al., 2011).

## Discussion

Our findings demonstrate that automated two‐way digital communication to improve medication adherence can target four different medication taking behaviors. While most interventions aimed to influence the behavior *taking medication*, the behavior *obtaining medication* had a greater association with improved medication adherence outcomes. *Obtaining medication* was targeted using a range of BCTs, and further research is needed to verify which BCTs are more or less effective.

The BCT most linked to results of increased medication adherence was *Problem solving* targeting *taking medication*. This BCT may facilitate the reduction of physical and/or psychological capability barriers to *taking medication*. Interventions which provided feedback on *taking medication*, even if this was conditional on identifying medication nonadherence, also seemed to be associated with improved medication adherence study results. This may also be due to the delivery of some form of problem solving, even if we could not code for this directly using the available descriptions. A recent meta‐regression review has also found that patient participants ‘reporting whether or not [taking medication] was performed’ was positively associated with increases in intervention effect size for medication adherence in cardio‐metabolic medicines (Kassavou & Sutton, 2018), although they did not look at feedback on this separately.

We found that interventions providing feedback on health outcomes from *taking medication* could also be used to increase reflective motivation for medication taking, and this has been suggested in another review (Mistry et al., 2015). However, these outcomes were rarely linked directly to medicine taking, and therefore patients may not be encouraged to make a direct connection between *taking medicines* and health outcomes. Qualitative studies with patient participants have found that evaluating outcomes of medication taking may be an important motivator for medication adherence (Kassavou, Houghton, Edwards, Brimicombe, & Sutton, 2019; Rathbone, Jamie, Todd, & Husband, 2020). However, providing feedback on outcomes may be complicated by the impact of other variables, such as lifestyle choices.

We found that providing one‐way messages delivering the BCT *Information about health consequences* did not seem to have a beneficial effect on medication adherence outcomes, although this is something which has been suggested as an effective strategy by others with cardio‐metabolic medicines (Kassavou & Sutton, 2017). Perceived necessity for medication has been found to be an important predictor of medication adherence (Horne & Weinman, 1999), and is something which could be influenced using this BCT. Several validated self‐report medication adherence tools incorporate a component of perceived necessity; however, as these are reported as composite scores, it was not possible to determine if changing medication perceptions was an outcome separate to overall adherence. Only one study measured this separately using the Beliefs about Medicines Questionnaire (BMQ) tool (Horne, Weinman, & Hankins, 1999), and this study did find an improvement in positive medication beliefs as a result of their intervention (Bender et al., 2010). However, as this was used alongside a range of other BCTs, how best to persuade patients of the beneficial outcomes from *taking medication* is something that requires further investigation.

*Prompts/Cues* aimed at *taking medication* was one of the most common BCTs included in digital communication interventions; however, we found that it did not seem to be connected to improving medication adherence outcomes. Other reviews evaluating digital communication reminders to improve medication adherence have found mixed results with some reporting a positive impact (Kannisto, Koivunen, & Välimaki, 2014; Sarabi et al., 2016; Tran, Coffman, Sumino, & Cabana, 2014) and others questioning their value (Free, Phillips, & Galli, 2013; Jörntén‐Karlsson, Pintat, Molloy‐Bland, Berg, & Ahlqvist, 2016; Mistry et al., 2015). Most of these reviews do not define what they consider to be a reminder, which could be one of several different BCTs from the taxonomy. One recent study allowed patient participants to choose whether they received reminders, (Nelson et al., 2020) and this might offer a better solution than routine incorporation of *Prompts/Cues*.

The model used to integrate automated two‐way digital communication with non‐digital intervention components varied among the included studies. Where there was the option of ‘live’ communication with healthcare professionals, we identified that SMS and IVR can be used to target the behavior *asking for support* for medication taking. We found no evidence that targeting the behavior *asking for support* had any impact on medication adherence or clinical outcomes in our review, but it was unclear what support was provided once patient participants sought additional help. In an article by Chiang, Guo, Amico, Atkins, & Lester (2018) eight additional BCTs were identified based on the ‘live’ interaction which followed from patient participants responding to a two‐way digital communication intervention. Other reviews have suggested that digital interventions should be supplemented with non‐digital components (Ciciriello et al., 2013; Fenerty et al., 2012; Granger & Bosworth, 2011; Hamine et al., 2015; Mistry et al., 2015). More explicit exploration of how non‐digital intervention components contribute to overall effectiveness of interventions alongside automated two‐way digital communication is an area for further research.

The behavior *obtaining medication* as a distinct behavior from *taking medication* has been identified in another review on digital communication to support medication adherence (Kassavou & Sutton, 2018). However, our behavioral map extends the list of behaviors that interventions can target to include *self‐testing* and *asking for support* with medicines taking. While other reviews have examined whether automated two‐way digital communication is effective for supporting adherence to medicines (Hamine et al., 2015; Kassavou & Sutton, 2018; Mistry et al., 2015), and others have included consideration of BCTs (Kassavou & Sutton, 2018; Long, Bartlett, Farmer, & French, 2019; Patton, Hughes, Cadogan, & Ryan, 2017), we are the first to extend this to how the delivery of such BCTs can influence patients’ capability, opportunity, and motivation to take their medication for long‐term conditions.

Delivery of the BCTs which were found to be related to improvements in outcomes required two‐way communication with the patient, and this is something that other reviewers have highlighted as important for digital communication intervention efficacy (Fjeldsoe et al., 2009; Granger & Bosworth, 2011; Kassavou & Sutton, 2018; Wald et al., 2015). A recent RCT examining one‐way text messaging found no improvement in medication adherence for medicines used in secondary prevention of cardiovascular disease, further supporting this conclusion (Bermon, Uribe‐Rodríguez, & Pérez‐Rivero, 2019). However, some BCTs are also more difficult to deliver in this clinical context, such as providing feedback on the outcomes of behavior. Guidance on designing text messaging programmes for health behavior change also suggests two‐way messaging to promote engagement in interventions (Abroms, Whittaker, Free, Mendel Van Alstyne, & Schindler‐Ruwisch, 2015).

Other reviews have questioned the potential for automated digital communication to be successful at improving medication adherence (Mistry et al., 2015); however, our analysis suggests this is not necessarily a barrier. This review has helped identify potential behavioral components to be used in a future intervention using automated two‐way text messaging to improve medication taking. Similar interventions are also in development, but have not chosen the same BCTs highlghted here as potentially helpful (Bartlett, Farmer, Rea, & French, 2020; Kassavou et al., 2019). The findings of such studies will help shed further light on the validity of our conclusions.

We focused on older forms of technology (SMS and IVR). While some newer technologies, such as smart phone apps, may offer the opportunity to deliver a wider range of BCTs, any potential for these needs to be offset against the reduced accessibility of this technology, for example, requirement for an internet connection and more expensive handset.

### Limitations

A limitation of our findings is that where behaviors are targeted in combination, the influence of each is not possible to separate. For example, some studies measure medication adherence using dispensing data, which are a measure of *obtaining medication* and not necessarily *taking medication*. A wide range of medication adherence measures were used within the included studies. Some of these were collected *via* the two‐way digital communication intervention and as such have not been validated. Most measures of medication adherence also only provide an indirect measurement of medication consumption (Lam & Fresco, 2015).

The lack of access to all intervention content also presents a previously identified challenge to the BCT coding process (Free, Phillips, & Watson, 2013; Pfaeffli Dale, Dobson, Whittaker, & Maddison, 2016). This was the case, in particular, when examining multiple studies using the same text messaging content, but where different example text messages were reported (Mayberry et al., 2017; Nelson, Mulvaney, Gebretsadik, Johnson, et al., 2016; Osborn & Mulvaney, 2013). We used guidance in the the BCW to assess how the included BCTs seemed to influence medication taking behaviors as these descriptions were mostly lacking within the articles themsleves. More explicit descriptions about inclusion of BCTs and their intended mechanisms would help future synthesis, and an ontology of behavior change intervention mechanisms has recently been created which should facilitate this (Marques, Carey, & Michie, 2021). Inability to compare findings across different studies has previously been highlighted as a limitation of research in the area of mobile health (Aranda‐Jan, Mohutsiwa‐Dibe, & Loukanova, 2014). We have also not covered in this review the potential role of tailoring interventions which could also mediate potential effectiveness of BCTs and has been highlighted by others (Fjeldsoe et al., 2009; Nundy et al., 2014; Vervloet et al., 2012).

### Areas for future research

More recently, the potential for mobile technologies to increase habit strength for medication taking has been highlighted (Badawy, Shah, Heneghan, & Beg, 2020); however only three studies were coded in this review for including the *Habit formation* BCT. All three had positive outcomes, but further research is needed to see how automated two‐way digital communication could further increase habit strength for *taking medication*, in particular, the frequency and length of contact required to affect this behavior in the longer term. Future research should also measure habit strength directly using tools, such as the Self‐Reported Habit Index (Verplanken & Orbell, 2013), to find out whether this is the mechanism by which providing feedback on behavior may improve medication adherence outcomes, and this is something which has also been highlighted by others (Badawy et al., 2020).

Only two studies included in this review targeted patient participants with more than one LTC. The growing prevalence of multimorbidity presents a major challenge for automated digital communication interventions, with or without non‐digital intervention components. It is also unclear which healthcare professional group may be best placed to accompany digital communication interventions, and this will need to be explored alongside the role that they would play in increasing motivation or removing barriers to medication taking.

Future research should also examine the contexts which allow behavioral mechanisms for automated two‐way digital communication interventions to work most effectively. This will add to our understanding about which patients should be offered these interventions, and in what circumstances. This would enable healthcare professionals to make informed decisions about what types of medication‐related support patients need. This is important as one type of intervention will not be appropriate for everyone.

### Conclusion

Automated two‐way digital communication, such as SMS and IVR, have already been shown to have positive effects on medication adherence and be acceptable to patient participants. This article provides some explanation as to why two‐way digital communication, with or without non‐digital components, may be more effective than one‐way communication. This was achieved by identifying the BCTs which can be delivered using this delivery format and the medication‐related behaviors which can be targeted. This includes the use of digital communication to monitor performance of the *taking medication* behavior in order to detect barriers, such as those relating to physical or psychological capability. Using digital communication to support the monitoring of outcomes from the behavior *taking medication* may also work to increase reflective motivation for behavior performance. Removing physical opportunity as a barrier to *taking medication* by targeting the pre‐requisite behavior, *obtaining medication*, may also be an important behavioral target, interventions where this is a barrier to medication adherence. Findings from this review can help inform the design of future automated two‐way digital communication interventions by suggesting behavioral elements that may support improved medication adherence for patients self‐managing their long‐term conditions.

## Conflict of interest

All authors declare no conflict of interest.

## Author contribution

**Gemma Donovan:** Conceptualization (equal); Formal analysis (equal); Funding acquisition (equal); Investigation (equal); Project administration (equal); Visualization (equal); Writing – original draft (equal); Writing – review & editing (equal). **Nicola Hall:** Formal analysis (equal); Investigation (equal); Writing – review & editing (equal). **Jonathan Ling:** Conceptualization (equal); Supervision (equal); Writing – review & editing (equal). **Felicity Smith:** Conceptualization (equal); Supervision (equal); Writing – review & editing (equal). **Scott Wilkes:** Conceptualization (equal); Funding acquisition (equal); Supervision (equal); Writing – review & editing (equal).

## Supporting information


**Appendix S1**. Search strategy, inclusion and exclusion crtieria.

**Appendix S2**. TIMELY data extraction form for narrative synthesis systematic review.

**Appendix S3**. TIMELY narrative synthesis behavior change coding manual.

**Appendix S4**. Quality appraisal of included studies using the Mixed Methods Appraisal Tool (MMAT) v1.

**Appendix S5**. Summary results table for the paper: Influencing medication taking behaviours using automated two‐way digital communication: A narrative synthesis.

## Data Availability

Data sharing not applicable – no new data generated.
