# Supplementary material for: Influencing medication taking behaviors using automated two‐way digital communication: A narrative synthesis systematic review informed by the Behavior Change Wheel
Source: Br J Health Psychol. 2022 Jan 26;27(3):861–90. doi: 10.1111/bjhp.12580 (PMC9541766; doi:10.1111/bjhp.12580)
Supplement: Supplementary file 1 — Appendix S1. Search strategy, inclusion and exclusion crtieria. [file BJHP-27-861-s005.docx]

**Influencing medication taking behaviours using automated two-way digital communication: A narrative synthesis systematic review informed by the Behaviour Change Wheel: Supplementary Document 1**

Databases searched

PubMed, Medline, CINAHL, PsycARTICLES, Psychology and Behavioural Sciences collection, Embase, International Pharmaceutical Abstracts, Web of Science and Cochrane Library. Grey literature was also searched including the Simple Telehealth Network, British Library EthOS, Trove and Opengrey.eu.

^7–10,12–14,22,25,27,30–38^

Example search strategy for EBSCO Medline

(telephone or cell phone or smartphone).ti.ab

(text messaging or reminder systems).ti.ab

(telemedicine or mobile health or telehealth or ehealth or mhealth).ti.ab

(text messag* or text-messag*).ti.ab

(short message service or SMS).ti.ab

(interactive voice response or IVR).ti.ab

(technology enabled care service or TECS).ti.ab

(automat* [within 3 words of] (land line or telephone or phone or call)).ti.ab

1 OR 2 OR 3 OR 4 OR 5 OR 6 OR 7 OR 8

(medication adherence or treatment refusal or patient compliance).mh

(medication adherence or treatment refusal or patient compliance).ti.ab

(compliance or adherence or persistence or concordance or nonadherence).ti.ab

(noncompliance or non-compliance or non-adherence).ti.ab

12 OR 13 (Medication$ or medicine$ or prescri* or therap*).ti.ab

13 [within 5 words of] 14

10 OR 11 OR 15

9 AND 16

Inclusion and exclusion criteria

| **Study aspect** | **Included** | **Excluded** |
| --- | --- | --- |
| Population | Adult patient participants (over 18 years) who were self-managing their medication in their own home with any long-term condition  High income countries as classified by the World Bank | Patients in care facilities e.g. care home or hospital |
| Intervention | Main intervention component should be:   - Two-way between the patient and the healthcare provider or research site - Automated in it’s delivery and/or response to incoming communication   Can include a non-digital communication component in addition to the main intervention | No additional exclusions |
| Comparator | Any | N/A |
| Outcomes | Study should report outcomes for medication adherence and/or clinical outcomes relevant to the long-term condition under study | No additional exclusions |
| Study type | Any | Pilot and feasibility studies |
| Other criteria | Published in English |  |
