# Supplementary material for: Influencing medication taking behaviors using automated two‐way digital communication: A narrative synthesis systematic review informed by the Behavior Change Wheel
Source: Br J Health Psychol. 2022 Jan 26;27(3):861–90. doi: 10.1111/bjhp.12580 (PMC9541766; doi:10.1111/bjhp.12580)
Supplement: Supplementary file 2 — Appendix S2. TIMELY data extraction form for narrative synthesis systematic review. [file BJHP-27-861-s001.pdf]

TIMELY Narrative Synthesis v2 Final

The data extraction form as part of the TIMELY Narrative Synthesis Systematic Review

*Required

1. Email *

2. Who is completing this data entry? *

Mark only one oval.

- Gemma
- Nicky

3. Citation for entry e.g. Donovan et al (2013) *

Study characteristics

4. Study design *

Tick all that apply.

- Randomised controlled trial
- Non-randomised controlled trial
- Cohort study
- Case-control study
- Cross-sectional analytic study
- Incidence or prevalence study without comparison group
- Case series
- Case report
- Questionnaire study
- Qualitative study
- Other:

5. If this was a comparative study with a control group, what was this? If multiple arms include details of each arm.

6. Select country of intervention *

Mark only one oval.

- Australia
- Austria
- Belgium
- Canada
- Chile
- China
- Croatia
- Cyprus
- Czech Republic
- Denmark
- Estonia
- Finland
- France
- Germany
- Greece
- Hungary
- Iceland
- Ireland
- Israel
- Italy
- Japan
- Korea, Rep.
- Latvia
- Lithuania
- Luxembourg
- Malta
- Monaco
- Netherlands
- New Zealand
- Norway
- Poland
- Portugal
- Puerto Rico
- Qatar
- Saudi Arabia
- Singapore
- Slovak Republic
- Slovenia
- Spain
- Sweden
- Switzerland
- United Arab Emirates
- United Kingdom
- United States
- Other

7. Date of study recruitment or "Not given" e.g. July 2010 *

____

8. Date of study close (until final follow-up) or "Not given" e.g. July 2011 *

9. Any other significant dates of interest? Please state date with description.

Participant characteristics

10. Number of participants included in the study or "Not given" *

11. Age of participants as Mean (SD) for total population or "Not given" *

12. Age of participants as Min-Max e.g. 18-24 or "Not given" *

13. What was the native language(s) of the participants? *

Tick all that apply.

- [ ] English
- [ ] French
- [ ] Spanish
- [ ] Unclear

Other: [ ]

14. Long term conditions included within the study *

Tick all that apply.

- [ ] Acne
- [ ] Anxiety disorders
- [ ] Asthma
- [ ] Atrial fibrillation
- [ ] Cancer
- [ ] Cardiovascular disease/ Ischaemic heart disease/ ACS/ MI/ Angina
- [ ] Chronic Obstructive Pulmonary Disease
- [ ] Dementia
- [ ] Depressive disorders
- [ ] Diabetes
- [ ] Epilepsy
- [ ] Glaucoma
- [ ] HIV/ AIDS
- [ ] Heart failure
- [ ] Hypertension
- [ ] Inflammatory bowel disease
- [ ] Organ transplantation
- [ ] Osteoporosis
- [ ] Pain (chronic)
- [ ] Parkinsons' dissease
- [ ] Psoriasis
- [ ] Rheumatoid arthritis
- [ ] Schizophrenia
- [ ] Stroke

Other: [ ] ____________________

15. Was the intervention targeted at any particular participant characteristic e.g. sociodemographic group? or "Not targeted" *

____
____
____
____
____

16. Was there any significant participant characteristic which was excluded e.g. patients with mental health problems? or "No significant exclusions" *

____
____
____
____
____

17. Percentage of females included in the study or "Not given" *

____

18. Any other information about the participant characteristics which may be relevant?

____

____

____

____

____

Intervention characteristics

19. Technology used *

Mark only one oval.

- ( ) IVR
- ( ) SMS
- ( ) Pager device
- ( ) Other: _______________

20. What language(s) was the intervention delivered in? *

Tick all that apply.

- [ ] English
- [ ] French
- [ ] Spanish
- [ ] Unclear

Other: [ ] _______________

21. Intended outcomes of the intervention in relation to medication adherence *

Tick all that apply.

- [ ] Promote medication adherence to a new therapy
- [ ] Maintain medication adherence to an established therapy
- [ ] Improve medication adherence to an established therapy
- [ ] To identify potential barriers to medication adherence
- [ ] To detect medication non-adherence
- [ ] Unclear

Other: [ ] _______________

22. Where there any additional intended outcomes of the interventior in relation to self-management of long term condition? For example improving knowledge, quality of life, self-efficacy, healthcare service utilisation or "No additional intended outcomes *

____

____

____

____

____

23. How long was the intervention? (Weeks/Months) or "Unclear" *

____

24. Did the intervention claim to be "tailored" or "personalised"? *

Tick all that apply.

- [ ] Personalised by using the patients' name in messages
- [ ] Personalised as patients' could choose when messages were sent
- [ ] Personalised as patient chose the message that they received e.g. designed the text
- [ ] Tailored to patients' identifiable barriers to medication adherence
- [ ] Tailored to patients' identifiable needs to facilitate self-care for their long term condition
- [ ] Describes tailoring or personalisation but unclear how this has been achieved
- [ ] No personalisation or tailoring

Other: [ ] ____________________

25. Did the intervention include a "reminder"? *

Tick all that apply.

- [ ] General reminder to adhere to medicines e.g. It is important to take your medicines every day
- [ ] Prompt reminder e.g. It's time to take your blood pressure medicines
- [ ] Monitoring reminder e.g. Did you take your blood pressure tablet today
- [ ] Yes, but it's unclear which type of reminder
- [ ] No reminder

26. How frequent was the communication intended to be e.g. daily, weekly or "Unclear frequency" *

____

27. How was the communication for the intervention initiated? *

Mark only one oval.

- ( ) Intervention initiated by the intervention deliverer e.g. healthcare profession, research team
- ( ) Intervention initiated by the patient e.g. by ringing a phone number or texting
- ( ) Intervention initiated by the intervention deliverer but patients could also initiate
- ( ) Intervention initiated by the patient but intervention could also initiate where patients do not respond
- ( ) A mixture of patient and intervention deliverer intiation
- ( ) Unclear

28. How frequently is the patient expected to interact with the intervention? e.g. Daily or "Unclear frequency" *

____

29. Are there any other technology elements included in the intervention? *

Tick all that apply.

- [ ] None
- [ ] Blood pressure monitoring
- [ ] Weight monitoring
- [ ] Pedometer
- [ ] Blood glucose monitoring
- [ ] Peak flow measurement
- [ ] Oxygen saturation
- [ ] Pulse monitoring

Other: [ ] ____________________

30. Are there any other additional communication components to the automated intervention? *

Tick all that apply.

- [ ] None
- [ ] Face-to-face consultations
- [ ] Website based content
- [ ] Synchronous telephone calls
- [ ] Videoconferencing
- [ ] Printed materials (please specify)

Other: [ ] ____________________

31. If printed materials were provided please provide details here

____

32. Were there any other non-communication components to the intervention e.g. creation of a shared management plan? *

____

33. To what extent is the content of the intervention available? *

Mark only one oval.

- ( ) Examples are provided
- ( ) The full content is available e.g. as a downloadable file
- ( ) Only a description of the content is available

34. Any other relevant information about the intervention design

____

____

____

____

____

Intervention delivery characteristics

35. Setting for sites involved in intervention delivery *

Tick all that apply.

- [ ] General practice
- [ ] Community based care e.g. Distric Nursing
- [ ] Out-patient care
- [ ] Community pharmacy
- [ ] Academic
- [ ] Non-health community setting
- [ ] Unclear

Other: [ ] ______________________

36. Number sites involved in intervention delivery or "Unclear" *

____

37. Software/ system used for automated patient contact delivery or "Unclear" *

____

38. Were sites remunerated for to cover the additional costs of the intervention compared to usual care? e.g. text messages, telephone calls *

Mark only one oval.

- ( ) Yes, fully
- ( ) Yes, partially
- ( ) No
- ( ) Not applicable
- ( ) Unclear

39. Were sites remunerated for the cost of the staffing time to delivei the intervention? *

Mark only one oval.

- ( ) Yes, fully
- ( ) Yes, partially
- ( ) No
- ( ) Not applicable
- ( ) Unclear

40. Were patients provided with equipment to participate in the intervention? e.g. mobile phone, pedometer *

Mark only one oval.

- Yes
- No
- Unclear

41. If equipment was provided, what was this?

____

42. Does the paper state that the costs for participants to communicate with the intervention were covered by the study? *

Mark only one oval.

- Yes
- No

43. 'Who' was communicating using the intervention *

Tick all that apply.

- [ ] Unclear
- [ ] Patients' own GP
- [ ] Patients' own primary care nurse
- [ ] Patients' own community pharmacist
- [ ] Specialist from secondary care (any profession)
- [ ] Researcher
- [ ] Persona based communication e.g. Flo

Other: [ ] ________________________

44. Any other relevant information about the intervention delivery

____

____

____

____

____

Outcomes of study

45. What were the intended outcomes of the study? *

____

____

____

____

____

46. Was clinical control measured as part of the study outcomes? e.g blood pressure *

Mark only one oval.

- Yes
- No *Skip to question 51*

Clinical Outcomes

47. What clinical outcomes were measured? e.g. blood pressue

____

48. At what time points was this measured? e.g. 0, 6 weeks

____

49. Did the study authors conclude that results support use of the intervention?

Mark only one oval.

- Yes
- No
- Unclear

50. Which outcome did they use to support this conclusion?

____

Outcomes of Study (Medication)

51. Was medication adherence measured as part of the study outcomes? *

Mark only one oval.

- Yes
- No *Skip to question 56*

Medication Adherence outcomes

52. How was medication adherence assessed?

Tick all that apply.

- [ ] Pill count
- [ ] Validated questionnaire tool e.g. Morisky
- [ ] Electronic container opening e.g. MEMS
- [ ] Medication supply data e.g. prescription refills
- [ ] Biochemical assay e.g. Drug level in blood
- [ ] Medication diary

Other: [ ] ________________________________

53. At what time points was this assessed? e.g. 0, 6 weeks

54. Did the study authors conclude that the results support use of the intervention?

Mark only one oval.

- Yes
- No

55. What outcome did they use to support this conclusion?

Study outcomes (patient acceptability)

56. Was patient acceptability assessed as part of the study outcomes? *

Mark only one oval.

- Yes
- No *Skip to question 59*

Patient Acceptability Outcomes

57. How was patient acceptability assessed?

Tick all that apply.

- Retention in the study e.g. not opting out
- Engagement in the study e.g. responding to text messages
- Quesionnaire
- Interviews
- Focus groups

Other:

58. Provide a short summary of their findings from assessing this

Study outcomes (professional acceptability)

59. Was professional acceptability assessed as part of the study outcomes?

Mark only one oval.

- Yes
- No *Skip to question 62*

Professional Acceptability Outcomes

60. How was professional acceptability assessed?

Tick all that apply.

- [ ] Retention in the study e.g. not opting out
- [ ] Engagement in the study e.g. number of patients signed up
- [ ] Quesionnaire
- [ ] Interviews
- [ ] Focus groups

Other: [ ] ____________________

61. Provide a short summary of their findings from assessing this

____
____
____
____
____

Study Quality Assessment

62. Is the study a mixed methods study incorporating qualitative and quantitative data? *

Mark only one oval.

- ( ) Yes
- ( ) No *Skip to question 67*

Mixed methods study

63. Is the mixed methods research design relevant to address the qualitative and quantitative research questions?

Mark only one oval.

- ( ) Yes
- ( ) No
- ( ) Can't tell

64. Is the integration of qualitative and quantitative data relevant to address the research question?

Mark only one oval.

- ( ) Yes
- ( ) No
- ( ) Can't tell

65. Is appropriate consideration given to the limitations associated with this integration?

Mark only one oval.

- ( ) Yes
- ( ) No
- ( ) Can't tell

66. Any comments on the mixed methods approach

____
____
____
____
____

Quantitative studies

Non-randomised study types include non-randomised controlled trials, cohort studies, case-control studies, cross-sectional analytic studies.

67. Does the study include a comparative element? *

Mark only one oval.

- ( ) Randomised controlled trial
- ( ) Non-randomised controlled trial *Skip to question 76*
- ( ) No comparative element *Skip to question 86*
- ( ) Other: ______________________

Quantitative (randomised controlled trial)

68. Provide a brief description of this data.

____
____
____
____
____

69. Is there a clear research question?

Mark only one oval.

- ( ) Yes
- ( ) No
- ( ) Can't tell

70. Do the collected data address the research question?

Mark only one oval.

- ( ) Yes
- ( ) No
- ( ) Can't tell

71. Is there a clear description of the randomisation?

Mark only one oval.

- Yes
- No
- Can't tell

72. Is there a clear description of the allocation concealment?

Mark only one oval.

- Yes
- No
- Not applicable
- Can't tell

73. Are there complete outcome data (80% or above)?

Mark only one oval.

- Yes
- No
- Can't tell

74. Is there a low withdrawal/drop-out (below 20%)?

Mark only one oval.

- Yes
- No
- Can't tell

75. Do you have any comments you wish to add?

____

____

____

____

____

Skip to question 86

Quantitative (non-randomised trial)

76. Provide a brief description of this data.

____

____

____

____

____

77. Is there a clear research question?

Mark only one oval.

- ◯ Yes
- ◯ No
- ◯ Can't tell

78. Do the collected data address the research question?

Mark only one oval.

- ◯ Yes
- ◯ No
- ◯ Can't tell

79. Are participants (organisations) recruited in a way that minimises selection bias?

Mark only one oval.

- ◯ Yes
- ◯ No
- ◯ Can't tell

80. Are measurements appropriate regarding the exposure/intervention and outcomes?

Mark only one oval.

- ◯ Yes
- ◯ No
- ◯ Can't tell

81. In the groups being compared, are the participants comparable, c do researchers take into account the difference between these groups?

Mark only one oval.

- ◯ Yes
- ◯ No
- ◯ Can't tell

82. Are there complete outcome data (80% or above)?

Mark only one oval.

- ◯ Yes
- ◯ No
- ◯ Can't tell

83. If applicable, is there an acceptable response rate (60% or above)

Mark only one oval.

- Yes
- No
- Can't tell
- Not applicable

84. If applicable, is there an acceptable follow-up rate for cohort studies?

Mark only one oval.

- Yes
- No
- Can't tell
- Not applicable

85. Do you have any comments you wish to add?

Quantitative studies

This includes prevalence studies, case series, case reports, questionnaire methods.

(descriptive)

86. Does the study have a quantitative descriptive element? *

Mark only one oval.

- Yes
- No *Skip to question 95*

Quantitative (descriptive)

87. Provide a brief description of this data.

88. Is there a clear research question?

Mark only one oval.

- Yes
- No
- Can't tell

89. Do the collected data address the research question?

Mark only one oval.

- Yes
- No
- Can't tell

90. Is the sampling strategy relevant to address the research question?

Mark only one oval.

- Yes
- No
- Can't tell

91. Is the sample representative of the population under study?

Mark only one oval.

- Yes
- No
- Can't tell

92. Are measurements appropriate?

Mark only one oval.

- Yes
- No
- Can't tell

93. Is there an acceptable response rate (60% or above)?

Mark only one oval.

- Yes
- No
- Can't tell

94. Do you have any comments you wish to add?

Qualitative studies

Includes ethnography, interviews, focus groups.

95. Does the study have a qualitative component? *

Mark only one oval.

- Yes *Skip to question 96*
- No

Qualitative study

96. Provide a brief description of this data.

____

____

____

____

____

97. Is there a clear research question?

Mark only one oval.

- Yes
- No
- Can't tell

98. Do the collected data address the research question?

Mark only one oval.

- Yes
- No
- Can't tell

99. Are the sources of qualitative data relevant to address the research question?

Mark only one oval.

- Yes
- No
- Can't tell

100. Is the process for analyzing qualitative data relevant to address the research question?

Mark only one oval.

- Yes
- No
- Can't tell

101. Is appropriate consideration given to how the findings relate to the context in which the data were collected?

Mark only one oval.

- Yes
- No
- Can't tell

102. Is appropriate consideration given to how the findings relate to researchers' influence through their interactions with participants?

Mark only one oval.

- Yes
- No
- Can't tell

103. Do you have any comments you wish to add?

This content is neither created nor endorsed by Google.

Google Forms
