# Supplementary material for: Influencing medication taking behaviors using automated two‐way digital communication: A narrative synthesis systematic review informed by the Behavior Change Wheel
Source: Br J Health Psychol. 2022 Jan 26;27(3):861–90. doi: 10.1111/bjhp.12580 (PMC9541766; doi:10.1111/bjhp.12580)
Supplement: Supplementary file 3 — Appendix S3. TIMELY narrative synthesis behavior change coding manual. [file BJHP-27-861-s002.docx]

**TIMELY Narrative Synthesis Coding Manual**

The BCT Taxonomy (v1) was used as a framework for coding, however further detail on coding decisions when applied to the TIMELY narrative synthesis is outlined in this manual.

Steps for coding:

1. Identify BCT according to BCT Taxonomy v1
2. Identify behaviour which BCT is targeting
3. Identify delivery mode for the BCT

Further guidance on coding BCTs for TIMELY Narrative Synthesis

- The description of the intervention delivery component should match definitions outlined in the BCT Taxonomy v1
- Where the delivery of a component could be one of two depending on a set of conditions, code both BCTs e.g. patient receives feedback if data input is considered ‘undesirable’ such as medication nonadherence but there is no feedback where patient is adherent, code both ‘Monitoring of behaviour without feedback’ and ‘Feedback on behaviour’. Where monitoring is forwarded to a human as part of the wider intervention, but no information is provided about use of this for delivering feedback, code only monitoring without feedback
- Where patient receives information about the body, code Biofeedback in addition to monitoring of outcomes of behaviour with or without feedback, or self-monitoring of outcomes of behaviour. Where outcomes are monitored and these require information to be provided by an external device, code Biofeedback, even if this is not explicitly described by the intervention.
- Self-monitoring of outcomes of behaviour will only be coded where the description of the intervention indicates that the patient is self-monitoring independently of the digital communication intervention, and using this self-monitoring to respond to digital queries. Self-testing and communicating results via the digital communication component is not considered to be self-monitoring of outcomes of behaviour, as we consider that the digital communication component is carrying out the ‘monitoring’ function rather than the patient
- The receipt of communication to trigger the performance of a behaviour is considered to be a prompt/ cue
- Where authors describe the consequences of medication taking in terms of either health outcome changes or experience of side effects, code for ‘Information about health consequences’. Where the intervention also seeks to provide reassurance around side effects for the purposes of reducing anxiety associated with fear of side effects code ‘reduce negative emotions’
- Provision of information relating to medicines and long-term conditions e.g. the long term condition the medicine is being used for, or the pathology of the disease itself, is not codable to a BCT. Where the authors describe the potential benefits of medicines taking on health, code to ‘Information about health consequences’, if the intervention explains how to take a medicine, code ‘Instruction on how to perform a behaviour’.
- ‘Verbal persuasion about capability’ will not be coded where the intervention only delivers generic encouragement e.g. "We know managing diabetes can be hard, but you can do it!"
- Use of the phrase ‘positive reinforcement’ will not be coded to any BCT without an explicit description of an intervention allowing the coding according to the BCT taxonomy v1

Further guidance on identifying behaviour associated with BCT coding

- Four behaviours have been identified *a priori* to be associated with medication adherence; obtaining medication, taking medication, self-testing and asking for support
- Obtaining medication should be coded for behaviours which lead to the possession of medication. This will usually be collecting medication from a pharmacy/ dispenser and/or requesting medication from a prescriber
- Taking medication is any behaviour which involves the consumption of a medication in any form e.g. solid oral dosage forms, inhalers, eye drops, topical products
- Self-testing refers to the use of at-home testing equipment which provides information about the body e.g. blood glucose monitors, blood pressure monitors
- Asking for support should be coded where patients contact another actor for the purposes of seeking support, which could be in the form of requesting further information, advice or practical help to support the other medication related behaviours
- Biofeedback, communication of results of self-testing (outcomes) and any subsequent feedback (or not) are coded the behaviour of medicines taking
- If patient receives contact from a healthcare professional as a result of reporting ‘undesirable’ results, code to relevant BCT for medication taking, not ‘Asking for support’.

Further guidance on identifying delivery mode for the BCT

- If the BCT is delivered via the automated digital communication component of the intervention (SMS, IVR or pager) then code to this delivery mode
- If the BCT is delivered outside of the automated digital communication component then code as part of the wider intervention
- If another actor contacts the patient as a result of communicating via the digital communication component, this interaction is considered as part of the wider intervention
- Self-testing as a behaviour, and any biofeedback resulting from this testing, takes place outside of the automated digital communication component. However, digital communication resulting from self-testing is coded depending on the presence or absence of feedback:
  - If results are inputted by patients, and no feedback is received, this is coded to the digital communication component
  - If feedback is delivered by the digital intervention, code to the digital intervention
  - If feedback is delivered by another actor, as a result of input via the digital communication component, code to the wider intervention
- Social support (unspecified) can only be delivered by a human as part of the wider intervention, the digital communication itself will not be considered as social support (unspecified)
- Examples of delivery mode which may be identified within wider intervention include mail, carers, and healthcare professionals

BCT and behaviours not considered within remit

Any BCTs delivered to target the patients’ engagement with the automated digital communication intervention itself should not be coded. This includes ‘Adding objects to the environment’ where digital devices are provided e.g. phones/ pagers. Monitoring associated with study procedures was also not coded for behavioural components. The automated digital communication intervention itself is not coded at social support BCTs.

Other behaviours targeted by the intervention such as those relating to diet and physical activity should not be coded, these are outside the remit of the review.
