# Supplementary material for: Influencing medication taking behaviors using automated two‐way digital communication: A narrative synthesis systematic review informed by the Behavior Change Wheel
Source: Br J Health Psychol. 2022 Jan 26;27(3):861–90. doi: 10.1111/bjhp.12580 (PMC9541766; doi:10.1111/bjhp.12580)
Supplement: Supplementary file 4 — Appendix S4. Quality appraisal of included studies using the Mixed Methods Appraisal Tool (MMAT) v1. [file BJHP-27-861-s004.docx]

Supplementary Document: Quality appraisal of included studies using the Mixed Methods Appraisal Tool (MMAT) v1*

| **Author (Date)** | **Primary study design** | **Secondary methods (where applicable)** | **MMAT Rating** |
| --- | --- | --- | --- |
| Bender et al (2010) | Randomised controlled trial | N/A | **** |
| Johnston et al [45] | Randomised controlled trial | N/A | **** |
| Tucker et al (2013) | Cohort study | N/A | **** |
| Piette et al (2000) | Randomised controlled trial | N/A | **** |
| Pfaeffli Dale et al (2015) | Randomised controlled trial | N/A | **** |
| Vollmer et al (2014) | Randomised controlled trial | N/A | **** |
| Magid et al (2011) | Randomised controlled trial | N/A | **** |
| Garofalo et al (2016) | Randomised controlled trial | N/A | *** |
| King et al (2017) | Cohort study | N/A | *** |
| Park et al (2014) and Park et al (2015) | Randomised controlled trial | N/A | *** |
| Mayberry et al (2017) | Cohort study | N/A | *** |
| Nelson et al (2016) | Case-control study | N/A | *** |
| Leu et al (2005) | Randomised controlled trial | N/A | *** |
| Aikens et al (2014) | Non-randomised controlled trial | N/A | *** |
| Aikens et al (2015) | Non-randomised controlled trial | N/A | *** |
| Nundy et al (2014) | Cohort study | Interviews to assess provider acceptability | *** |
| Zabinski et al (2012) | Cohort study | N/A | *** |
| Katelenich et al (2015) | Randomised controlled trial | N/A | *** |
| Wald et al (2014) | Randomised controlled trial | N/A | *** |
| Spoelstra et al (2016) | Randomised controlled trial | N/A | *** |
| Bove et al (2013) | Randomised controlled trial | N/A | *** |
| Sherrard et al (2015) | Randomised controlled trial | N/A | *** |
| Glanz et al (2012) | Randomised controlled trial | N/A | *** |
| Sherrard et al (2009) | Randomised controlled trial | N/A | *** |
| Piette et al (2015) | Randomised controlled trial | N/A | *** |
| Friedman et al (1996) | Randomised controlled trial | N/A | *** |
| Boker et al (2012) | Randomised controlled trial | N/A | ** |
| Harris et al (2010); Simoni et al (2010); Yard et al (2011) | Randomised controlled trial | Focus group and questionnaire assessing patient acceptability | ** |
| Kuusalo et al. [44] | Randomised controlled trial | Questionnaire to assess patient acceptability | ** |
| Moore et al (2015) | Randomised controlled trial | N/A | ** |
| Stacy et al (2009) | Randomised controlled trial | N/A | ** |
| Cizmic et al (2015) | Randomised controlled trial | N/A | ** |
| Vollmer et al (2011) | Randomised controlled trial | N/A | * |
| Shane-McWhorter et al (2014) | Cohort study | N/A | * |
| Boland et al (2014) | Randomised controlled trial | N/A | * |
| Stuart et al (2003) | Randomised controlled trial | N/A | - |

*Note that this study was conducted before the introduction of the MMAT v2 which was published in 2018
